# Design and rationale of the social determinants of the risk of hypertension in women of reproductive age (SAFE HEART) study: An American Heart Association research goes red initiative

**DOI:** 10.1016/j.ahj.2024.05.016

**Published:** 2024-06-09

**Authors:** Faith E. Metlock, Yaa A. Kwapong, Crystal Evans, Pamela Ouyang, Dhananjay Vaidya, Ebenezer Kobbie Aryee, Khurram Nasir, Laxmi S. Mehta, Roger S. Blumenthal, Pamela S. Douglas, Jennifer Hall, Yvonne Commodore-Mensah, Garima Sharma

**Affiliations:** a Johns Hopkins School of Nursing, Baltimore, MD; b Johns Hopkins Bloomberg School of Public Health, Baltimore, MD; c Johns Hopkins Ciccarone Center for the Prevention of Cardiovascular Disease, Baltimore, MD; d Houston Methodist, Houston, TX; e The Ohio State Wexner Medical Center, Columbus, OH; f Duke Clinical Research Institute, Duke School of Medicine, Durham, NC; g American Heart Association, Dallas, TX; h Inova Health System, Falls Church, VA; i Institute of Clinical and Translational Research, Johns Hopkins University School of Medicine; j Johns Hopkins School of Medicine, Baltimore, MD

## Abstract

**Background:**

Cardiovascular health literacy (CVHL) and social determinants of health (SDoH) play interconnected and critical roles in shaping cardiovascular health (CVH) outcomes. However, awareness of CVH risk has declined markedly, from 65% of women being aware that cardiovascular disease (CVD) is the leading cause of death for women in 2009 to just 44% being aware in 2019. The American Heart Association Research Goes Red (RGR) initiative seeks to develop an open-source, longitudinal, dynamic registry that will help women to be aware of and participate in research studies, and to learn about CVD prevention. We proposed to leverage this platform, particularly among Black and Hispanic women of reproductive age, to address CVHL gaps and advance health equity.

**Methods:**

The primary objective of the study is to evaluate the cross-sectional association of CVHL, SDoH using a polysocial score, and CVH in women of reproductive age at increased risk of developing hypertension (HTN). To achieve this we will use a cross-sectional study design, that engages women already enrolled in the RGR registry (registry-enrolled). To enhance the racial and ethnic/social economic diversity of the cohort, we will additionally enroll 300 women from the Baltimore and Washington D.C. community into the Social Determinants of the Risk of Hypertension in Women of Reproductive Age (SAFE HEART) Study. Community-enrolled and registry-enrolled women will undergo baseline social phenotyping including detailed SDoH questionnaire, CVH metrics assessment, and CVHL assessment. The secondary objective is to assess whether a 4-month active health education intervention will result in a change in CVHL in the 300 community-enrolled women.

**Discussion:**

The SAFE HEART study examines the association between CVHL, SDoH, and CVH, with a focus on racial and ethnic minority groups and socioeconomically disadvantaged women of reproductive age, and the ability to improve these parameters by an educational intervention. These findings will inform the future development of community-engaged strategies that address CVHL and SDoH among women of reproductive age.

## Background

Despite significant efforts over recent decades to improve public awareness that cardiovascular disease (CVD) is the leading cause of death for US women, awareness declined markedly, from 65% of women being aware that CVD is the leading cause of death for women in 2009 to 44% being aware in 2019.^1^ This decline in awareness is particularly alarming among women of reproductive age, as they face increased risks of adverse cardiovascular events during pregnancy, including hypertensive disorders such as preeclampsia and gestational hypertension (HTN). These conditions not only impact maternal health but also pose serious risks to fetal well-being. Therefore, examining cardiovascular health literacy (CVHL) and interconnected social risks among women of reproductive age is crucial for identifying strategies to improve maternal and fetal health outcomes.

CVHL, refers to the understanding and application of health information specifically related to preventing, managing, and treating CVD. It involves not only comprehending medical information but also translating that knowledge into health-promoting behaviors and actions. For instance, individuals with high CVHL may better understand the importance of diet and exercise in managing HTN and be more likely to adhere to prescribed medications and lifestyle recommendations. Considering the significant role of health literacy in both primary and secondary prevention of CVD,^2^ it is essential to investigate CVHL as a distinct subset of health literacy. Currently, there are notable gaps in research specifically linking health literacy to HTN, with few studies measuring it as a predictor or covariate. Incorporating health literacy measurements in prospective community-based studies may faciliatate exploration of its impact on HTN and blood pressure management.^2^ Additionally, including health literacy assessments in cardiovascular clinical trials may shed light on its influence on treatment adherence and enhance the applicability of findings to individuals with limited health literacy, who are often underrepresented in such studies.^2^

Understanding CVHL is essential for addressing health disparities and improving CVH outcomes. Individuals with limited CVHL often face barriers to accessing preventive care, managing chronic conditions, and navigating the healthcare system. These barriers are often intertwined with social determinants of health (SDoH), which are “the conditions in the environments where people are born, live, learn, work, play, worship, and age that affect a wide range of health, functioning, and quality-of-life outcomes and risk.”^3^ These factors include income, education, housing, and access to healthcare resources. Within the intersecting SDoH domains, there are multilevel (eg, individual, community, and policy) factors shaped by the distribution of money, power, and resources that contribute significantly to health inequities.^4,5^ Racial and ethnic minorities, who are disproportionately impacted by adverse SDoH factors such as limited education attainment and lower socioeconomic status, experience higher rates of limited health literacy.^6,7^ These factors influence CVH outcomes, including HTN prevention and management.^8–10^

The progress of understanding women’s specific risks, symptoms, diagnosis, and treatment in cardiovascular research has been hindered by inadequate representation of women in research studies.^11–13^ Recognizing this gap, the American Heart Association (AHA) established the Research Goes Red (RGR) platform to address the under-representation of women in clinical trials.^14^ The RGR initiative seeks to develop an open-source, longitudinal, dynamic registry that will allow women to be aware of and participate in research studies, and to learn about CVD prevention. Though over 15,000 individuals have engaged with RGR, self-identified non-Hispanic White are over-represented at 75.7% and Black women are under-represented at 10.5%.^14,15^ Further, the majority of participants are of higher socioeconomic status, which limits the ability to generalize findings from this registry to the US population. Results from studies that feature homogenous samples of participants may fail to show the full variability in responses that may occur when the full heterogenous population is included.^12,13^

Meaningful efforts to increase the representation of groups disproportionatly impacted by limited CVHL, adverse SDoH, and suboptimal CVH in research requires use of community-engagment principles.^16–19^ In accordance with the AHA’s priority to diversify the RGR registry in order to address CVH gaps and advance health equity, we propose to leverage the infrastructure already developed for this platform while increasing inclusion of Black and Hispanic Women of reproductive age into the registry through community-based recruitment.^20–22^ Using a cross-sectional and quasi-experimental study design, our study plans to explore the association between CVHL and SDoH on CVH among women of reproductive age. By leveraging community engagement principles, we seek to involve community groups as key stakeholders in our study.

## Methods

### Study objective and design

Using a cross-sectional study design, our study examines the association between CVHL, SDoH, and CVH among women of reproductive age who are at increased risk for developing HTN. We will enroll 300 participants already participating in the RGR registry (registry-enrolled group). To broaden the ethnic/social economic diversity of the cohort, we will additionally enroll 300 women from the Baltimore and Washington D.C. community. These community-enrolled women will also be enrolled into a quasi-experimental study, the Social Determinants of the Risk of Hypertension in Women of Reproductive Age (SAFE HEART) Study. Community-enrolled and registry-enrolled women will undergo baseline social phenotyping including detailed SDoH questionnaire, CVH metrics assessment, and a CVHL assessment.

The primary objective (Aim 1) of the cross-sectional study is to evaluate the association of CVHL, SDoH, and CVH, in women of reproductive age at increased risk of developing HTN. We hypothesize that women with higher aggregate polysocial scores^23^ (ie, with more adverse SDoH) will be at greater risk of HTN. We postulate that more of these women will have suboptimal CVH (defined as *≥*2 cardiovascular risk factors based on Life’s Essential 8^24^) compared to women with low polysocial scores, a greater proportion of whom will have optimal CVH (*≤*1 Life’s Essential 8 cardiovascular risk factor). We further postulate that women with poor CVHL will have suboptimal CVH.

**Figure 1.**
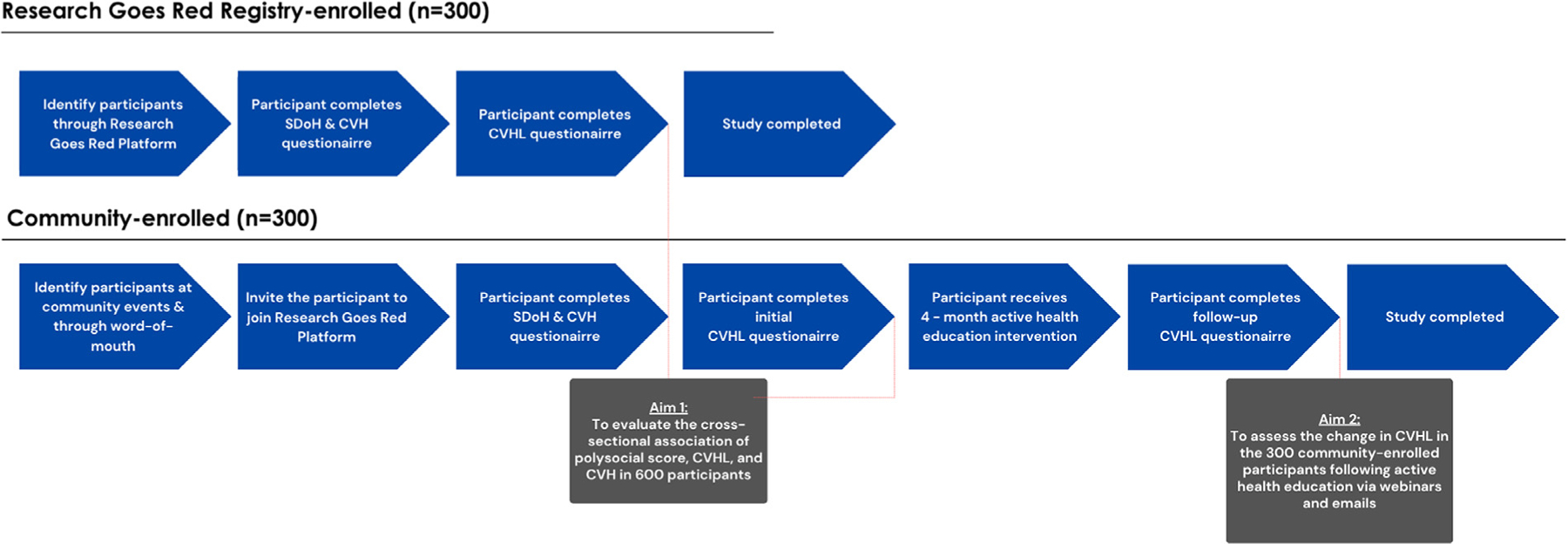
Study flowchart.

The SAFE HEART Study will evaluate whether CVHL will improve following a 4-month active health education intervention (Aim 2). The 300 community-enrolled participants enrolled from the Baltimore/Washington DC area will undergo an active 4-month health education intervention with patient- centered materials including recorded videos, presentations and modules. Performance on the CVHL assessement will be compared before and after the educational intervention (Figure 1). We hypothesize that the CVHL of participants will improve over a period of 4 months. This study has been approved by the Johns Hopkins Medicine Institutional Review Board.

### Conceptual framework

**Figure 2.**
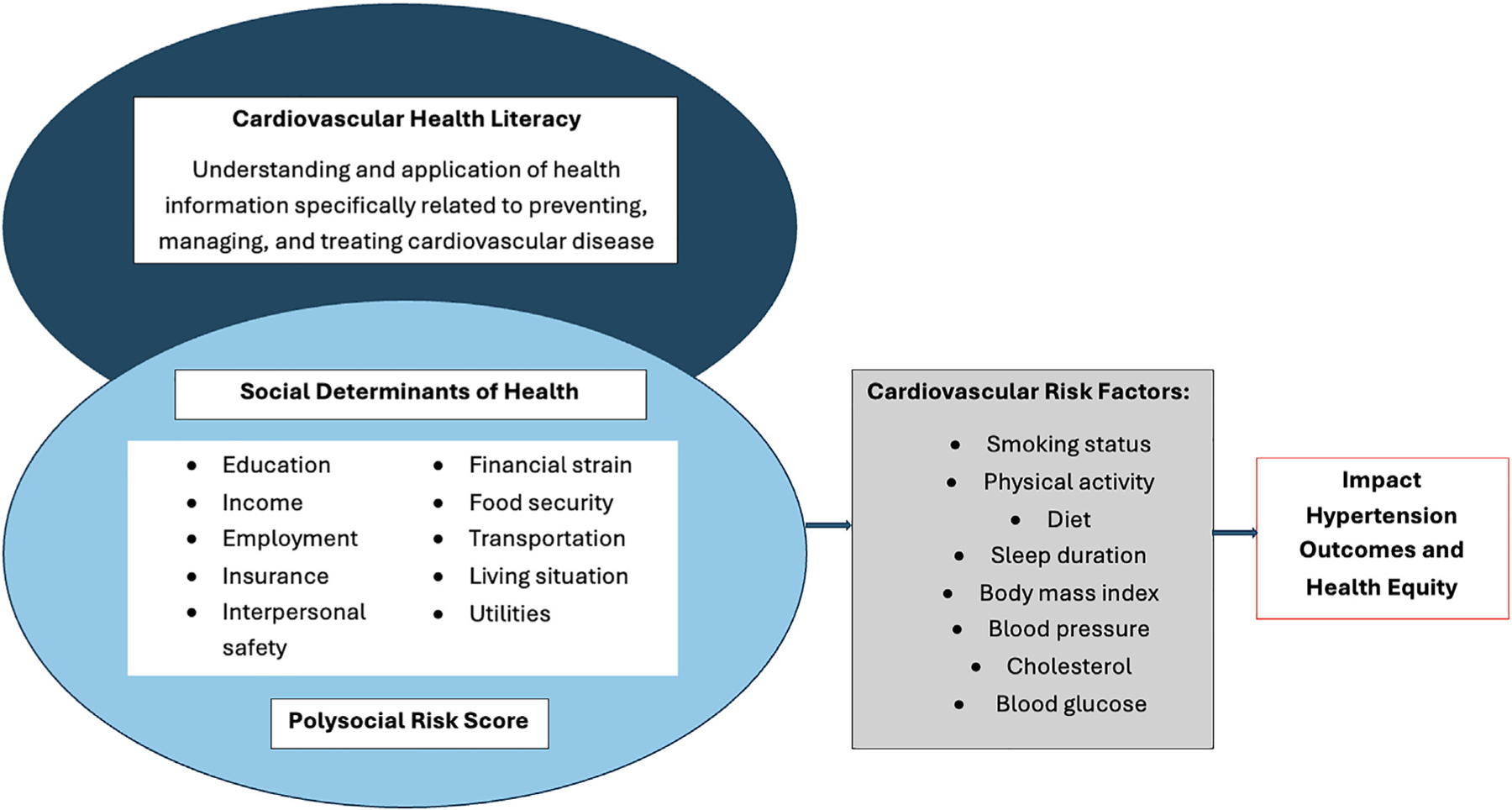
Adapted conceptual framework.

The Critical Framework of Social Determinants of Health,^25–27^ and Health Literacy and Social Determinants framework^2^ have been adapted to guide this study. CVHL and SDoH are interconnected with exposure to adverse SDoH potentially affecting CVH risk factors such as blood pressure, leading to disparities in HTN outcomes.^28^ CVHL is measured using the Heart Disease Facts Questionaire (HDFQ). We capture SDoH factors using sociodemographic information and the Accountable Health Communities Health-Related Social Needs screening tool. Biological and psychological sequalae are examined via self-report of cardiovascular risk factors including smoking status, physical activity, healthy diet, body mass index (BMI using height and weight), blood pressure, cholesterol levels, blood glucose levels, and sleep duration (Figure 2).

### Research variables and measurement

#### HDFQ

We will utilize the HDFQ^29^ to assess CVHL among participants. The HDFQ is a validated 25-item questionnaire designed to gauge respondents’ knowledge of major risk factors for coronary heart disease, with a particular focus on diabetes-related coronary heart disease risk factors. The questionnaire is comprehensible to the average 13-year-old, imposes minimal burden on participants while demonstrating good content and face validity. Internal consistency is adequate, with a Kuder–Richardson-20 formula of 0.77, and item-total correlations are favorable.^29^

Respondents are asked to indicate whether each statement is “true,” “false,” or “I don’t know,” with 6 items scored in reverse. Correct responses are scored as “1,” while incorrect responses or “I don’t know” are scored as “0.” The total score, ranging from “0 – 100,” is obtained by multiplying the number of correct responses by 4. Based on scoring thresholds, total scores falling below 50 are categorized as “poor,” and scores *≥*50 as “adequate.” Additionally, the 25 items are further categorized to assess knowledge across different domains^30^: 1) role of age, gender, genetics and family history, 2) risk factors for CVD, 3) role of exercise in prevention, 4) role of diet and cholesterol levels in CVD, and 5) role of therapeutic measures and lifestyle intervention for CVD (Table S1).

### Accountable health communities health-related social needs (AHC-HRSN) tool

The SDoH measures included in the study are derived from sociodemographic information and questions from the AHC-HRSN tool. Created by the Centers for Medicare & Medicaid Services, the AHC-HRSN is a 10-item screening tool to identify patient needs that can be addressed through community services in 5 core domains (housing instability, food insecurity, transportation problems, utility help needs, and interpersonal safety). Supplemental questions from additional domains are also available (eg, financial strain).^31^

#### Polysocial risk

Using a cumulative social disadvantage approach, a polysocial risk score^5^ will be quantified to assess SDoH. A total of 14 SDoH from 7 distinct domains will be evaluated: 1) sociodemographic information 2) living situation, 3) food security, 4) transportation, 5) utilities, 6) safety, and 7) financial strain. The individual questions used to record information on each SDoH in the original survey, and the operational definitions used in this study to classify each SDoH as “favorable” or “unfavorable” are listed in Table S2. The 14 identified SDoH will be assigned each a value of “1” if unfavorable (eg, uninsured), and “0” if favorable (eg, insured). An aggregate SDoH burden in the population will be calculated by summing the 14 individual SDoH, with a resulting range of 0 to 14.

#### Life’s essential 8

In our study, CVH will be assessed using the Life’s Essential 8 framework,^24^ which comprises 8 key factors influencing CVH. These factors include smoking status, physical activity, diet, body mass index (BMI), blood pressure, cholesterol levels, blood glucose levels, and sleep duration. Each factor will be scored as “1” for unfavorable outcomes and “0” for favorable outcomes, based on established criteria. The total score will provide an overall assessment of CVH, with higher scores indicating poorer CVH. Suboptimal CVH will be defined as having “2” or more CVD risk factors, while optimal health will be defined as having “0” or “‘1” CVD risk factor.

### Community-enrolled participants

#### Study setting

This community-based intervention study is being conducted in the Baltimore metropolitan area. The city of Baltimore is racially diverse, 64.0% of the population is Black, 29.4%.

Non-Hispanic White, and 6.6% other races. In this study, we will leverage our established relationships with community-based organizations and faith-based organizations to enroll the participants.

#### Community engagement

We developed a dynamic partnership with the Community Research Advisory Council (C-RAC) of the Johns Hopkins Institute for Clinical Research (ICTR) Community Collaboration Core (CCC), which is made up of 22 members representing patients/research participants, community-based organizations, neighborhood associations, health systems, and historically Black colleges and universities.^32^ The community research advisory board was created to address health equity issues among Maryland residents. To meaningfully commit to genuine community engagement, we met virtually with the advisory council during the conceptualization and recruitment phase. Based on their recommendations, we have implemented valuable recruitment strategies.

During the prefunding stage, C-RAC assembled a focus group that consisted of 19 C-RAC members, 4 reproductive-age women with a history of high blood pressure, and 1 family caregiver for a person with high blood pressure. During the session, the principal investigator (G.S.) asked the participants impacted by high blood pressure for recommendations on ‘how to engage women of reproductive age who are at risk of developing high blood pressure.’ One participant stated that ‘some of us are not good at going to the doctors or staying on top of our health unless there is a known risk factor.’ So, participants suggested that study materials be disseminated at daycare/early education facilities, community health centers, and health fairs.

The study team meets with the C-RAC every year to present updates on outreach, recruitment, and dissemination, as well as to solicit feedback on study priorities. To execute the C-RAC suggestions, and achieve its study objectives the study team employed a community coordinator (C.E.) to act as a liaison between the research team and the Baltimore community. The Community Coordinator has been crucial in establishing linkages between our research team and local community outreach events. We have also established important ties with faith-based organizations in the Baltimore area. Our team attends monthly events to convey important information about CVH in women using AHA materials (eg, booklets, infographics), provide blood pressure checks, and identify possible study participants. This frequent interaction helps establish our presence in the community and fosters trust, which is consistent with our commitment to community-driven research.

#### Recruitment

**Figure 3.**
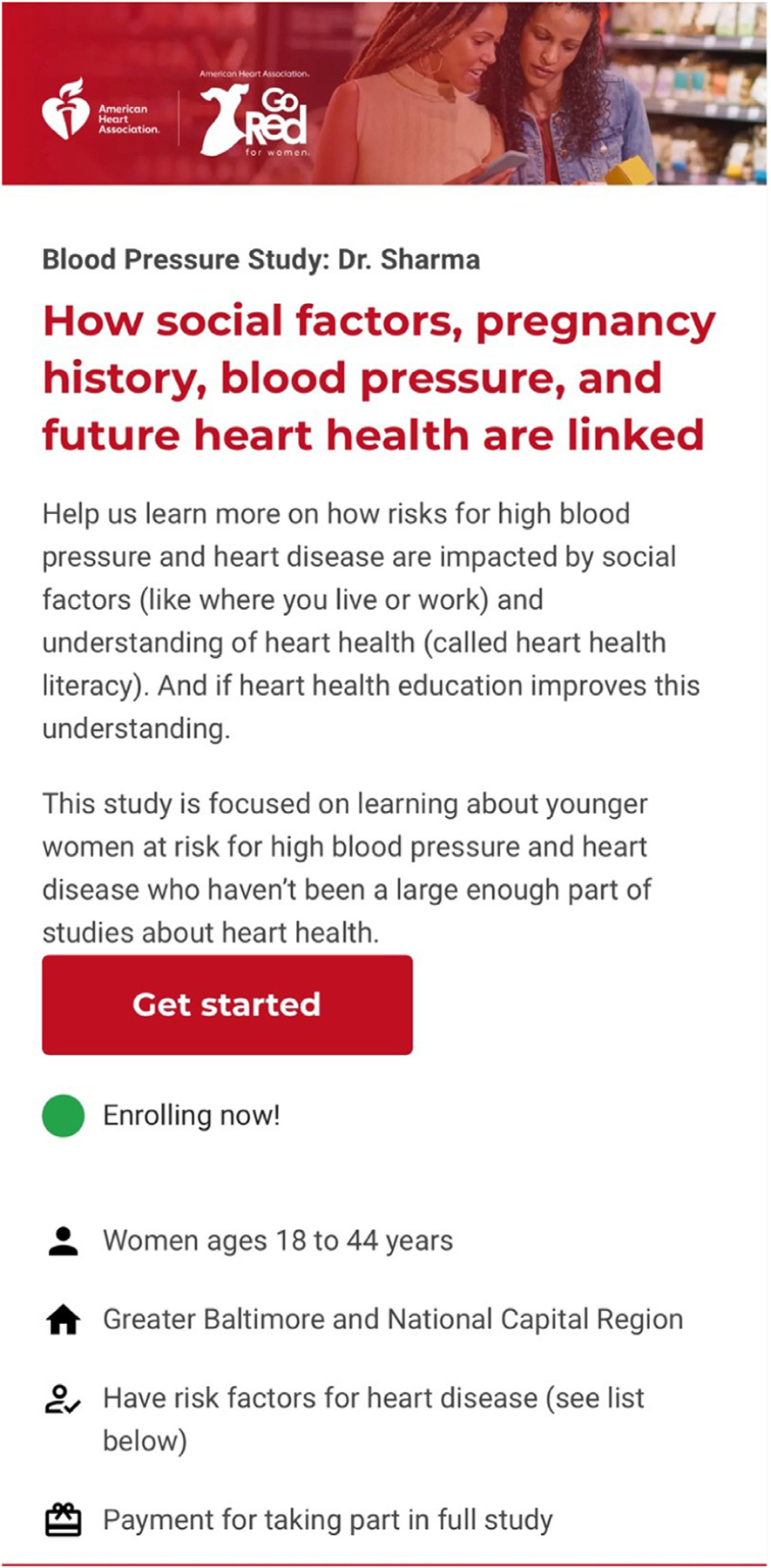
Study landing page.

Our recruitment strategy is to approach women of reproductive age residing in the Baltimore metropolitan area based on the criteria in Table 1. Participants are identified primarily at community health events and through word-of-mouth. Interested individuals are then screened based on study criteria. Eligible participants are consented and then encouraged to immediately sign up on the online RGR platform using the participant’s smartphone, or the study team provided iPad. Participants may also take time to consider joining the study and can wait to e-consent and sign up to the RGR platform until they are ready using a provided website landing page (Figure 3). The study information and landing page will also be advertised via flyers located at frequented community areas. A participant is considered enrolled once they have provided e-consent and completed study baseline surveys in RGR.

#### Intervention

All community-recruited participants will receive an active health education program aimed at promoting CVHL. This education initiative features the dissemination of monthly newsletters developed by the study team. These newsletters emphasize the importance of mitigating CVD risk factors and offer supplementary learning resources from the AHA, such as recorded videos and instructional modules from the EmpOWERED to Serve initiative.^33^ Notably, these resources cover a range of topics including “Salt and Cardiovascular Risk,” “Make Your Life Sweet, Not Your Drink,” and “Eating Smart: Fruits and Vegetables.” To foster deeper engagement and understanding, our study team will also conduct monthly virtual webinars that correspond to the CVD risk factor themes featured in the monthly newsletters. Table 2 showcases the CVHL competencies. Each participant will be offered access to this active health education intervention for a duration of 4 months.

#### Newsletters

The newsletters are designed and disseminated through the Microsoft Sway platform. Each CVD risk factor being addressed that month emphasizes relevant statistics and highlights disparities to provide a comprehensive understanding of the issue for women’s health. Following the problem identification, the next section in the newsletter pivots to explain why addressing the featured CVD risk factor is vital for overall CVH. This segment highlights the benefits of optimizing CVD risk factor while also outlining the potential consequences of neglect. The selected CVD risk factor section concludes action-focused by offering practical guidance on how participants can take control of their CVH through healthier behaviors. Drawing from the AHA resources, this section provides actionable steps and strategies to improve CVD risk factors. Finally, the newsletters undergo evaluation for readability at a 5th-grade level using online tools, ensuring accessibility.

#### AHA resources

Our active health education intervention leverages a robust set of resources from the AHA EmpOWERED to Serve initiative.^33^ This initiative aligns well with the focus of our study as it is grounded in the belief that every individual deserves an equitable opportunity to achieve optimal health, regardless of their social position or systemic challenges that contribute to health disparities. The publicly available health lessons include 13 science-based toolkits, spanning 2 critical domains: health education and community advocacy. The resources cover a wide array of CVH subjects, from promoting smoke-free communities to understanding family health history, managing conditions during COVID-19, and controlling blood pressure. These resources also address critical topics such as nutrition, heart health, and CPR training, underscoring the holistic approach to improving CVH and overall wellbeing.

### Registry-enrolled participants

#### Recruitment

Our recruitment strategy involves identifying individuals in the RGR database whose age and metropolitan area characteristics match those of community-enrolled participants. We plan to oversample and contact 400 participants through the RGR platform who meet these criteria. Invitations will be extended to this subset, encouraging them to take part in our study surveys.

The registry-enrolled participants will not receive compensation due to the platform’s limitations in supporting these capabilities. Unlike the community-enrolled participants, these individuals will not undergo the active health education intervention, and no follow-up assessments will be conducted as part of the study.

#### Study time frame

This study is being carried out over 2 years. In year 1, the study team will formalize collaborations with key stakeholders including community and faith-based organizations, apply for IRB approval, finalize study protocol, and train study personnel, among other study start-up activities. Activities in year 2 include patient recruitment and enrollment, intervention implementation, and data collection. Community-enrolled participants will receive 4 months of the active health education intervention. At the end of year 2 the team will complete the follow-up data collection, conduct data analysis, and disseminate the findings.

#### Outcomes

**Figure 4.**
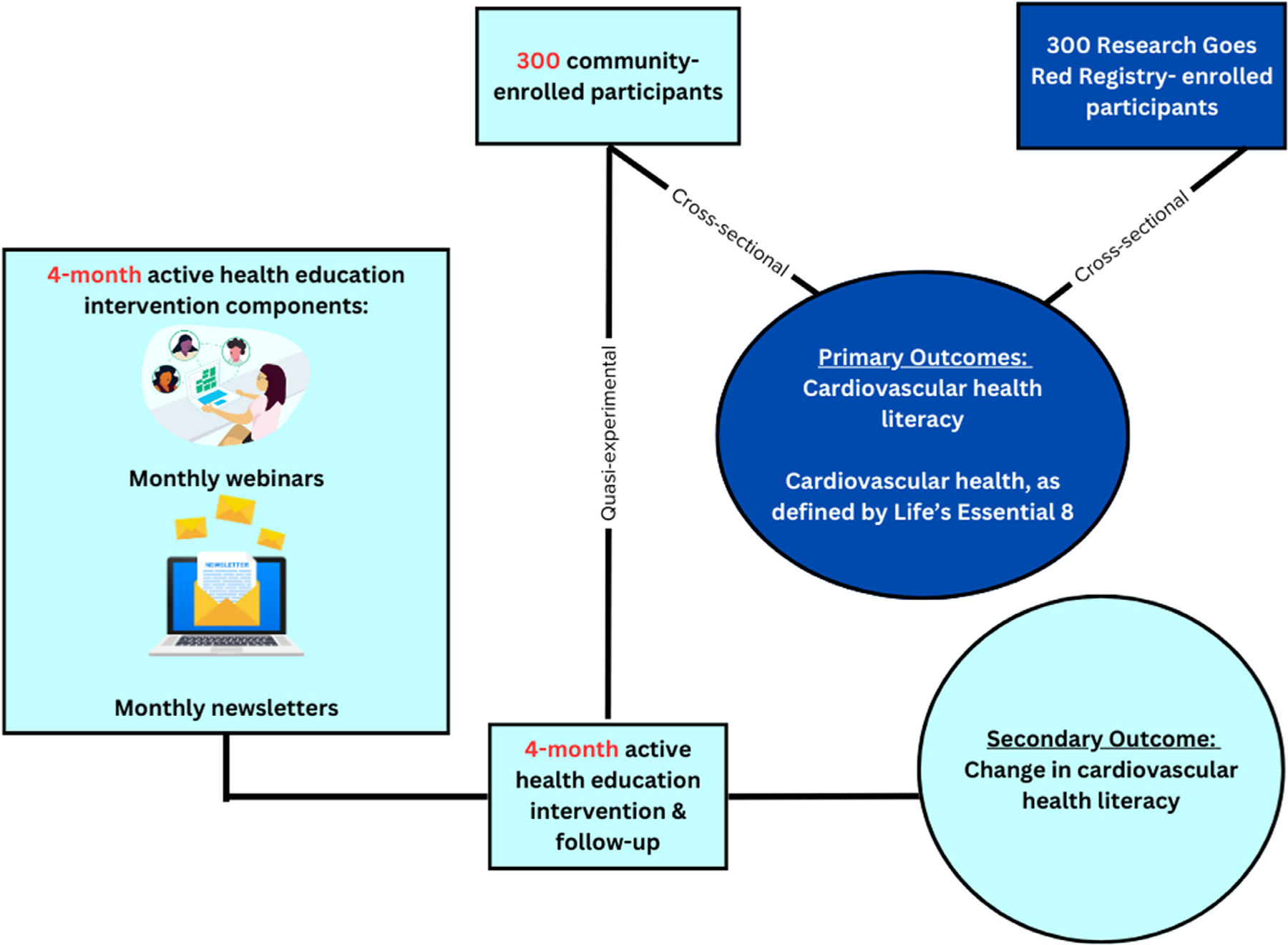
Study outcomes.

The primary outcomes of this study are CVHL and CVH. The secondary outcome is change in CVHL from baseline to 4 months in the community-enrolled participants exposed to the educational intervention (Figure 4).

### Statistical analysis plan

Prior to outcome analysis, we will perform descriptive analyses of our sample including distributions of demographic, clinical and measurements including suboptimal CVH and CVHL levels. For categorical variables we will present numbers and percentages by category, and for continuous variables, we will present means and standard deviations for normally distributed variables and medians and interquartile ranges for non-normally distributed variables. We will perform exploratory bivariate analyses between demographic and measured variables to guide possible covariable adjustment.

For Aim 1, our primary outcome variables are CVHL and CVH. We will preliminarily graphically examine the bivariable association of CVHL and CVH as continuous dependent variables with polysocial score as the independent variable. Based on the preliminary analyses we will dichotomize CVHL (poor and adequate) and CVH (optimal and suboptimal CVH). We will examine associations using tests of differences in proportions. Based on the exploratory bivariable analyses for covariates, we will perform demographic variable adjusted analysis using logistic regression analyses.

For Aim 2, the outcome is the 4-month longitudinal change in CVHL. We will first examine the distribution of the change in health literacy in our study participants and tabulate the changes by demographic groups. We will use longitudinal mixed model analysis with CVHL as the dependent variables and polysocial risk score as the independent variable. A difference in changes in CVHL by the baseline polysocial risk score levels will be tested in terms of an interaction term between time and the baseline levels of polysocial score.

### Power calculation

For Aim 1 we are presenting the size of the 95% precision range for incident optimal CVH literacy, and the minimum detectable effect size with 80% power at the p *<*0.05 level (MDE80) in the difference in incident optimal CVHL between those with polysocial score below versus above median. Assuming that suboptimal CVH literacy prevalence at baseline is 90%, 75% or 50%; the 95% precision of the estimate of incident optimal CVH at baseline is *±*6, 7 and 8%, respectively. lllustrating a mid-optimistic scenario of an overall incident optimal CVH is 50%, the MDE80 for the difference of incidence in low versus high polysocial score in the 3 scenarios of baseline prevalences is 17%, 19%, and 23%, respectively.

For Aim 2, we have 80% power to detect a difference in longitudinal improvements in health literacy between below median-SDoH women versus above median-SDoH women of 0.2 standard deviation units of health literacy (assuming moderate repeat measurement correlation of 0.5) or 0.14 standard deviation units of health literacy (assuming high repeat measurement correlation of 0.8). In addition, this sample size is adequate to detect a cross sectional correlation of 0.11 at baseline between polysocial score and CVHL.

## Results

To date, our study team has participated in 14 community health events hosted at several locations including community centers, parks, food markets, and faith-based organizations. Of the 217 participants enrolled, 68 were directly recruited at these community health events, while the majority (149) were recruited through word-of-mouth referrals by community members encountered at these gatherings (Table 3). Through these means of reaching the community, our data thus far indicates an enrichment of Black women participants in the racial and ethnic breakdown. Regarding our active health education intervention, we have shared monthly newsletters with our participants on the following topics: Month 1) “Control Your Blood Pressure,” “Get Active,” and “Eating Smart with Fruits and Vegetables;” Month 2) “Know Diabetes by Heart,” “Keeping a Healthy Body Weight;”: Month 3) “Understanding My Cholesterol Risk,” “Create Smoke-Free Communities,” and “How Sleep Affects Your Health.” We are finishing up recruitment of community-enrolled participants and preparing to start recruitment of RGR-enrolled participants.

## Discussion

Our study focuses on the association between CVHL, SDoH and CVH among women of reproductive age, with a particular focus on women from minority racial and ethnic groups. Health literacy plays a pivotal role in an individual’s ability to access healthcare information and make informed decisions. Specifically, CVHL is crucial for understanding and managing cardiovascular risk factors and engaging in self-care behaviors aimed at improving CVH. This study measures CVHL among a community disproportionately burdended by CVD risk factors, who often grapple with adverse social factors such as limited education and socioeconomic status. These SDoH significantly impact health outcomes and perpetuate health inequities, particularly among HTN prevention and control.

CVD remains a substantial threat to women’s health, with declining awareness over the past decade.^1^ The under-representation of women, particularly women of color, in clinical trials poses a significant barrier to advancing our understanding of women’s specific CVD risks, symptoms, diagnosis, and treatment. The RGR initiative aims to rectify this issue by promoting research opportunities for women and providing a longitudinal dynamic registry for research studies. However, the current participant composition in RGR demonstrates a significant lack of representation, with most participants identifying as non-Hispanic White, and a lack of representation from socioeconomically disadvantaged groups.^14^ This lack of diversity hinders the generalizability of research findings and calls for concerted efforts, grounded in community-engaged principles, to enhance representation among vulnerable populations.

Our study’s exploration of the interplay between CVHL, SDoH, and CVH among women of reproductive age holds significant promise in understanding the heightened cardiovascular risk faced by nonwhite women at an age where intervention to reduce risk may have significant benefit. Our findings will help illuminate the importance of including a diverse sample of individuals in clinical research in order to understand the population-level impact of therapies. Furthermore, this study will provide important information on the role of disparities and CVHL as determinants of CVH. Through our study, we aim to contribute to a growing body of evidence that advocates for comprehensive community-engaged approaches to maternal health that prioritize the unique needs and challenges faced by women.

The study’s setting in the racially diverse city of Baltimore provides an opportunity to engage with a community facing varying socioeconomic and health challenges. Leveraging established relationships with community-based and faith-based organizations has been instrumental in participant recruitment and engagement. By participating in outreach events and sharing valuable information on CVH with hopes of promoting lifestyle changes, we establish a presence within the community, fostering trust and reinforcing our commitment to community-engaged research.^34^

Our study’s active health education intervention, featuring monthly newsletters and virtual webinars, has been designed to empower participants with CVHL. These resources draw from the AHA’s EmpOWERED to Serve initiative, offering science-based toolkits and health lessons that cover a wide range of CVH topics. By offering actionable steps and strategies, we aim to promote healthier behaviors and empower participants to take control of their CVH. While our current study is limited to English-language materials due to constraints within the RGR registry, we acknowledge the significance of ensuring materials are accessible to individuals for whom English is not their first language. In future nested studies, we plan to translate materials into Spanish to ensure broader inclusivity.

Our intervention is notable for its multifaceted community-engaged approach to addressing CVH disparities among populations made vulnerable. Unlike previous studies, we have developed a comprehensive set of educational materials tailored to the needs and priorities of young women in Baltimore and Washington, D.C. These materials, including monthly newsletters and virtual webinars conducted in a group format, are designed to be visually appealing, interactive, and accessible. Importantly, our webinars provide a platform for participants to engage in discussion with cardiovascular health professionals, fostering community support and enhancing the impact of the intervention. We have ensured the relevance and resonance of our intervention within the target population by actively involving community members and organizations in its development and dissemination. Our collaboration with community and faith-based organizations, engagement with the advisory councils, and dedication to community engaged principles underscore our commitment to advancing CVH among underserved communities. By exploring the relationship between CVHL, SDoH, and CVH, we aim to contribute to the body of knowledge that informs interventions promoting primordial and primary prevention of CVD through community-engagement. Ultimately, our study seeks to empower women of reproductive age to make informed decisions about their CVH, reducing disparities and improving overall health outcomes.

### Early lessons in engaging women of reproductive age in community-based efforts

Engaging women of reproductive age in community-based cardiovascular health research necessitates a multifaceted approach grounded in community engagement principles. Here, we share early insights into community engagement strategies that have proven to be successful in the recruitment and retention of participants thus far.

1. Acknowledging community as an identity: The cornerstone of our engagement strategy involves meeting potential participants in settings where they naturally congregate and their environments. This approach includes regular presence at local markets, faith-based organizations, and community health events. Through these encounters, we’ve established a distinct study identity marked by branded materials, including tablecloths featuring AHA’s Go Red for Women branding. Additionally, the incorporation of engaging study branding elements, such as customized logos featured on our promotional materials, has played a pivotal role in elevating our study’s visibility and appeal. By adopting a professional and visually appealing aesthetic, our study branding not only conveys credibility but also ensures that our efforts are memorable and easy to recognize. This approach has effectively set us apart, allowing us to stand out in the community, attract increased interest, and forge stronger connections with potential participants who might otherwise overlook our outreach endeavors. Moreover, giveaways, including items with AHA branding such as water bottles and drawstring bags, have been made available to foster engagement with our study table and team.
2. Fostering co-learning and capacity building: Building trust and heightening awareness of CVD has been central to our approach, with recruitment being viewed as an organic byproduct of our outreach efforts. This includes providing blood pressure screenings, regardless of recruitment interest, and prioritizing open dialogues to address community health concerns. We have also recognized the significance of team composition, emphasizing diversity within our study team. Our inclusion of a Community Relations Coordinator team member from the Baltimore community has facilitated an in-depth understanding of local barriers and facilitators, enhancing trust among community members.
3. Building on strengths and resources of the community: Notably, our experiences highlight that word-of-mouth promotion and survey interest forms have proven more effective in recruitment than community health events, which tend to attract an older demographic. While this approach has significantly broadened our reach, it has also introduced challenges related to potential fraudulent accounts. Unlike participants enrolled at community health events, individuals recruited through word-of-mouth methods present a verification hurdle, as we cannot immediately confirm their study eligibility. To mitigate this limitation, we implemented a strategic solution: requiring individuals enrolled through word-of-mouth to join our monthly zoom webinar calls. This postenrollment verification step ensures that we can authenticate participant information before compensating, promoting a more reliable recruitment process.
4. Balancing knowledge and intervention for mutual benefit for all partners: In response to valuable feedback from our community members and partners, we have refined our compensation approach. Originally, we offered a total compensation of $25 upon the completion of all follow-up surveys. However, driven by a commitment to better serve our participants, we decided to increase the compensation by now awarding $25 Amazon claim codes incrementally, providing participants with a total of $50 for survey completion. This adaptation not only acknowledges the time and effort invested by our participants more comprehensively but also reflects our responsiveness to community input, ensuring that the compensation structure aligns with the expectations and preferences of our valued participants.

These initial insights from the SAFE HEART study offer valuable guidance for researchers seeking to engage women of reproductive age in community-based research, emphasizing that trust-building and community awareness are paramount in the early phases of such endeavors.

## Supplementary Material

SAFEHEARTPROTOCOL_Supp

## Figures and Tables

**Table 1.** Inclusion/exclusion criteria.

Inclusion	Exclusion
Between the age of 18 to 44 y old	Age *<* 18 and *>* 44 y old
Self-identify gender as a woman	Diagnosis of end-stage renal disease (ESRD)
Have elevated BP (120–129/ *<* 80 mm Hg) and Stage 1 hypertension based on the ACC/AHA Hypertension guidelines, or	Condition which interferes with outcome measurement (eg, dialysis)
Previous pregnancy with hypertensive disorder of pregnancy, or	Serious medical condition which either limits life expectancy or requires active management (eg, cancer)
Physical inactivity ( *<* 150 min of aerobic exercise a wk), or	Patients with cognitive impairment or other condition preventing their participation in the intervention
Obesity ( *≥*30 Kg/m^2^ ), or	Those with an active alcohol or substance use disorder (ie, not sober/abstinent for *≥*30 d)
Diagnosed with diabetes, or	Unwillingness to provide informed consent
Current smoker	
Able to speak and read English	

**Table 2.** Active health education competencies derived from American Heart Association’s EmPOWERED to serve initiative and other resources.

Topics	Objectives
Control your blood pressure	• Understand information about blood pressure and the normal range for blood pressure numbers Learn how high blood pressure can impact your health • Discover things you can do to help control blood pressure
Eating smart with fruits and vegetables	• Embrace ways to include a variety of fruits and vegetables in your daily meals to promote heart healthy eating • Learn ways to add more color, crunch and flavor with produce • Take action to help improve access to fruits and vegetables for everyone
Get active	• Understand the importance of physical activity guidelines for Americans’ cardiovascular health • Identify how to remove barriers to physical activity • Establish a physical activity plan
Know diabetes by heart	• Understand what diabetes is. • Know about its link to heart disease and stroke • Understand how to take charge of your health to reduce your risk
Create smoke-free communities	• Gather facts about the effects of tobacco use and impact on cardiovascular health. • Be an advocate for smoke-free communities
How sleep affects your health	• Learn how sleep is connected to heart disease and overall well-being • Realize steps to get better sleep • Establish a wake-up routine
Keeping a healthy body weight	• Recognize the relationship between weight and cardiovascular health • Understand when your weight is in a healthy range • Identify steps to lose weight and keep it off
Understanding my cholesterol risk	• Understand cholesterol levels and the American Heart Association’s recommendations • Discover lifestyle changes for the prevention and treatment of high cholesterol

**Table 3.** Recruitment method.

Recruitment method	*N* (%)
Community outreach events	68 (26.2)
Word-of-mouth referrals	149 (73.7)
