# Supplementary material for: Design and rationale of the social determinants of the risk of hypertension in women of reproductive age (SAFE HEART) study: An American Heart Association research goes red initiative
Source: Am Heart J. Author manuscript; Available in PMC 2025 Apr 28. (PMC12036016; doi:10.1016/j.ahj.2024.05.016)
Supplement: SAFEHEARTPROTOCOL_Supp [file NIHMS2071265-supplement-SAFEHEARTPROTOCOL_Supp.docx]

Design and Rationale of the Social Determinants of the Risk of Hypertension in Women of Reproductive Age (SAFE HEART) Study: An American Heart Association Research Goes Red Initiative

Faith E. Metlock, PhD_c_, RN^1^; Yaa A Kwapong, MD, MPH^3^; Crystal Evans^9^, MS; Pamela Ouyang, M.B.B.S^.9,^ FACC, FAHA; Dhananjay Vaidya, M.B.B.S., Ph.D., MPH.; Ebenezer Kobbie Aryee MBChB, MPH^;3^ Khurram Nasir, MD MS ^4^; Laxmi S. Mehta, MD ^5^; Roger S. Blumenthal, MD^3^; Pamela S. Douglas, MD ^6^;  Jennifer Hall, PhD^7^; Yvonne Commodore-Mensah, PhD, MPH, FAHA RN^1,2^; Garima Sharma, MD,^8,3^

^1^Johns Hopkins School of Nursing, Baltimore, MD, USA

^2^Johns Hopkins Bloomberg School of Public Health, Baltimore, MD, USA

^3^Johns Hopkins Ciccarone Center for the Prevention of Cardiovascular Disease, Baltimore, MD, USA

^4^ Houston Methodist, Houston, TX, USA

^5^ The Ohio State Wexner Medical Center, Columbus, OH, USA

^6^ Duke Department of Medicine, Durham, NC, USA

^7^ American Heart Association, Dallas, TX, USA

^8^Inova Health System, Falls Church, VA, USA

^9^Institute of Clinical and Translational Research, Johns Hopkins University School of Medicine

Corresponding Author:

Garima Sharma, MD, FACC, FAHA

Director, Women’s Cardiovascular Health and Cardio-obstetrics

Inova Health System, Falls Church, VA

Adjunct, Associate Professor of Medicine,

Johns Hopkins University School of Medicine

garima.sharma@inova.org

**Supplemental Materials:**

| **Table S1.** **Research Variables and Measurement** | |
| --- | --- |
| **Research Variable** | **Measurement outcomes** |
| **Dependent Variables** | |
| Cardiovascular health literacy:  Heart Disease Facts Questionairre^29^ | Correct responses are scored as "1," while incorrect responses or "I don't know" are scored as "0." The total score, ranging from "0 - 100," is obtained by multiplying the number of correct responses by four.  Poor CVHL = scores < 50  Adequate CVHL = ≥ 50 |
| Cardiovascular health:  Life’s Essential 8  Have you ever been told by a doctor that you had:   - Diabetes or high blood sugar - High cholesterol - High blood pressure   Have you smoked at least 100 cigarettes in your entire life?  Aside from your job, how many minutes of physical activity or exercise (such as running, walking, etc.) do you do per week?  Not including fruit juices, how many servings of fruits/vegetables do you consume per day?  How many hours of sleep do you get per night?  BMI: What is your weight & height? | Each factor will be scored as '1' for unfavorable outcomes and '0' for favorable outcomes. Suboptimal CVH= ≥ 2 risk factors & optimal health= ≤ 1 CVD risk factors  Dichotomous: yes and no  Dichotomous: yes and no  Dichotomous: 150 minutes or more and Less than 150 minutes  Dichotomous: 5 or more and less than 5  Categorical: 9 hours or more, 7-9 hours, Less than 7 hour  Continous: self-report value in pounds and feet and inches  BMI score ≤ 25 vs >25 |
| **Independent Variables** | |
| **Individual SDoH** | |
| **Sociodemographic information** | |
| **Education (1)**: What is the highest grade of formal or academic education have you had? | Categorical: some college – advanced degree, some high school – high school graduate, and less than high school. |
| **Employment (1):** We would like to know about what you do for work. For example, are you working full-time, working part-time, looking for work, retired, keeping house, or a student? | Categorical: employed, unemployed, and student |
| **Income (1):** What was the total combined income of your household in the past year? This can include income from wages, salaries, Social Security/retirement benefits, help from relatives, or other sources of income.  How many people are supported by this income right now? Count yourself and everyone supported by this income, even if they do not live in the same household as you. | Categorical: $150,000 or more, $100,000-$149,999, $50,000-$99,999, and less than $50,000 |
| **Marital Status (1):** What is your current marital status? | Categorical: single, married, cohabiting, and divorced or seperated |
| **Insurance (1):** Do you currently have health insurance? | Categorical: government-sponsored, private, and uninsured |
| **Home ownership (1):** Please select the best way to describe the home where you live | Categorical: Owned, Rented, Occupied without payment |
| **Accountable Health Communities Health-Related Social Needs tool^31^** | |
| **Living situation (2):**  What is your living situation today?  Think about the place you live. Do you have problems with any of the following? CHOOSE ALL THAT APPLY | Categorical: worried about losing housing, unsteady place to live, and steady place to live  Categorical: pests such as bugs, ants, or mice/mold/lead paint or pipes/lack of heat/oven or stove not working/smoke detectors missing or not working/water leaks/other and none of the above |
| **Food security (2):**  Within the past 12 months, you worried that your food would run out before you got money to buy more.  Within the past 12 months, the food you bought didn't last and you didn't have money to get more. | Categorical: often true, sometimes true and never true |
| **Transportation (1):** In the past 12 months, has lack of reliable transportation kept you from medical appointments, meetings, work or from getting things needed for daily living? | Dichotomous: yes and no |
| **Utilities (1):** In the past 12 months has the electric, gas, oil, or water company threatened to shut off services in your home? | Categorical: yes, already shut off and no |
| **Safety (1):**  How often does anyone, including family and friends, physically hurt you?  How often does anyone, including family and friends, insult or talk down to you?  How often does anyone, including family and friends, threaten you with harm?  How often does anyone, including family and friends, scream or curse at you? | Continuous: Never (0 points); rarely (1 point); sometimes (2 points); fairly often (3 points); frequently (5 points).  scores ≥ 11 vs. scores < 11 |
| **Financial strain (1):** How hard is it for you to pay for the very basics like food, housing, medical care, and heating? Would you say it is: | Categorical: very hard, somewhat hard, and not hard at all |
| **Cumulative SDoH** | |
| **Polysocial risk score:** A polysocial risk score will be calculated by summing the 14 individual SDoH. | Continous: Polysocial risk score ranging between 0-14  The 14 identified SDoH will be assigned each a value of of ‘1’ if unfavorable (e.g., uninsured), and ‘0’ if favorable (e.g., insured). Higher scores indicate more social risks. |
| Underline = unfavorable outcome | |

**Table S2. Heart Disease Facts Questionairre**

| **HEART DISEASE FACTS QUESTIONAIRRE** | | | | |
| --- | --- | --- | --- | --- |
|  |  | **True** | **False** | **I don’t know** |
| 1 | A person always knows when they have heart disease. |  |  |  |
| 2 | If you have a family history of heart disease, you are at risk for developing heart disease. |  |  |  |
|  |  |  |  |  |
| 3 | The older a person is, the greater their risk of having heart disease. |  |  |  |
| 4 | Smoking is a risk factor for heart disease. |  |  |  |
| 5 | A person who stops smoking will lower their risk of developing heart disease. |  |  |  |
| 6 | High blood pressure is a risk factor for heart disease. |  |  |  |
|  |  |  |  |  |
| 7 | Keeping blood pressure under control will reduce a person’s risk for developing heart disease |  |  |  |
|  |  |  |  |  |
| 8 | High cholesterol is a risk factor for developing heart disease. |  |  |  |
| 9 | Eating fatty foods does not affect blood cholesterol levels. |  |  |  |
| 10 | If your ‘good’ cholesterol (HDL) is high you are at risk for heart disease. |  |  |  |
| 11 | If your ‘bad’ cholesterol (LDL) is high you are at risk for heart disease. |  |  |  |
| 12 | Being overweight increases a person’s risk for heart disease. |  |  |  |
| 13 | Regular physical activity will lower a person’s chance of getting heart disease. |  |  |  |
| 14 | Only exercising at a gym or in an exercise class will lower a person’s chance of developing heart disease. |  |  |  |
| 15 | Walking and gardening are considered exercises that will help lower a person’s chance of developing heart disease. |  |  |  |
| 16 | Diabetes is a risk factor for developing heart disease. |  |  |  |
| 17 | High blood sugar puts a strain on the heart. |  |  |  |
| 18 | If your blood sugar is high over several months, it can cause your cholesterol level to go up and increase your risk of heart disease. |  |  |  |
| 19 | A person who has diabetes can reduce their risk of developing heart disease if they keep their blood sugar levels under control. |  |  |  |
| 20 | People with diabetes rarely have high cholesterol. |  |  |  |
| 21 | If a person has diabetes, keeping their cholesterol under control will help to lower their chance of developing heart disease |  |  |  |
| 22 | People with diabetes tend to have low HDL (good) cholesterol |  |  |  |
| 23 | A person who has diabetes can reduce their risk of developing heart disease if they keep their blood pressure under control |  |  |  |
| 24 | A person who has diabetes can reduce their risk of developing heart disease if they keep their weight under control |  |  |  |
| 25 | Men with diabetes have a higher risk of heart disease than women with diabetes |  |  |  |
| **Area of Knowledge** | | | **Items** | |
| Role of age, gender, genetics and family history | | | 1,2,3,25 | |
| Risk factors for CVD | | | 4,6,8,12,16,18 | |
| Role of exercise in prevention | | | 13,14,15 | |
| Role of diet and cholesterol levels in CVD | | | 9,10,11,17,20,22 | |
| Role of therapeutic measures and lifestyle intervention for CVD | | | 5,7,19,21,23,24 | |

**Figure S1. Newsletter Example**
